# The impact of training problem-solving skills on self-esteem and behavioral adjustment in teenage girls who have irresponsible parents or no parents 

**Published:** 2015

**Authors:** F Shahgholy Ghahfarokhi, N Moradi, P Alborzkouh, S Radmehr, M Zainali

**Affiliations:** * Clinical Psychology, Islamic Azad University, Science and Research Branch, Isfahan, Iran; ** Clinical Psychology, Islamic Azad University Karaj Branch, Iran; *** Exceptional Children Psychology, Islamic Azad University Central Tehran Branch, Iran; **** Clinical Psychology, Islamic Azad University Chaloos Branch, Iran; ***** General Psychology, Humanities and Social Sciences Faculty, Paradise University, Gillan Branch, Iran

**Keywords:** training problem-solving skills, self-esteem, behavioral adjustment, teenage girls with irresponsible parents or no parents

## Abstract

Proper psychological interventions are of great importance because they help enhancing psychological and public health in adolescents with irresponsible parents or no parents. The current research aimed to examine the impact of training problem-solving experiment on self-esteem and behavioral adjustment in teenage girls with irresponsible parents or no parents.

**Methodology:** The approach of the present research was a semi-test via a post-test-pre-test model and a check team. Hence, in Tehran, 40 girls with irresponsible parents or no parents were chosen by using the Convenience modeling, and they were classified into 2 teams: control and experiment. Both groups were pre-tested by using a demography questionnaire, Rosenberg’s self-esteem scale, and a behavioral adjustment questionnaire. Afterwards, both groups were post-tested, and the obtained data were examined by using inferential and descriptive methods through SPSS 21.

**Findings:** Findings indicated that the training problem-solving skills significantly increased the self-esteem and the behavioral adjustment in teenage girls with irresponsible parents or no parents (P < 0/ 001).

**Conclusion:** The conclusion of this research was that training problem-solving methods greatly helps endangered people such as teenage girls with irresponsible parents or no parents, because these methods are highly efficient especially when they are performed in groups, as they are cheap and accepted by different people.

## Introduction

According to WHO (World Health Organization) (2009) [**23**], adolescence includes ages between 11 and 19 years. People in this age group face significant physical, mental, and social changes. In addition, the pressure of this life stage causes mental disorders [**9**]; many mental disorders diagnosed at the age of 14 [**12**].

Today, the various evolutions on social bases, which are due to the movement from traditional lifestyles to industrial lifestyles, have caused gaps in humans’ lives, leading to numerous emotional, psychological, and social damages [**9**]. Hence, it is no surprise that family bases are weak, requiring houses for children and adolescents with no parents. In the 24-hour houses, there are children and young people of the following sorts: children whose parents were jailed because of drug abuse or drug trafficking, and children who escape their homes because of intra-family problems as well as cultural, economic, and ethical poverty (Ahmadi, 2001) [**22**]. In this age group, girls are more prone to danger. In a comparison between girls with no parents and normal girls, it was found that there are significant differences regarding depression, brainstorm, social incompatibilities, pessimism, withdrawal, and feeling insecure [**9**]. 

Nevertheless, one of the most influential parts protecting humans against anxiety, pressure, and stressful events is self-esteem. Self-esteem is an essential parameter, which can be associated with various factors in life, affecting humans’ observable behaviors [**24**]. Besides the fact that it includes the fourth need from Maslow’s hierarchy of needs, it supplements to personalities’ confidence and independence [**25**]. Findings collected from many investigations revealed that a weak self-esteem has outcomes such as distress and grief [**26**], physical and mental diseases [**9**], communicational and behavioral difficulties and corrupt actions [**26**]. In addition, this item requires a special attention especially when it comes to adolescents with irresponsible parents or no parents.

Also, one of the things which might be affected by the lack of social support is individuals’ social adjustment [**8**]. A social adjustment like physical, emotional, and rational growth is a continuous quantity which gradually becomes perfect [**5**]. Adolescents can determine their positions in their communications with peers and adults by using social skills to receive social acceptance [**17**]. In fact, “adjustment” refers to the communication between individuals and their environment (especially social environment); this connection enabling them to respond to their needs and motivations [**17**]. Therefore, training and interventions, which can enhance adjustment, are highly beneficial. 

Training life skills is very useful regarding the forming and improving the individuals’ abilities [**13**]. One of the most important skills is difficulty solving. As it is known, knowing and using a difficulty-solving experiment is a prerequisite for emotional treatment and performance regulating [**8**]. Training problem-solving skills is a therapeutic method through which individuals learn how to employ their cognitive experiments to cope via problematic interpersonal status [**5**]. Because individuals lacking social support might be prone to different psychological problems and to adjustment (Neusk, Hogez & Suiiden, 2003). Also, regarding the fact that one of the characteristics of inadaptable individuals is the lack of problem-solving skills [**18**], it is very important to improve this skill in people with little social support. As mentioned above, it is critical to do research to identify ways to increase adjustment and self-esteem in teenage girls with irresponsible parents or no parents.

## Methodology

The present research is semi-test with a post-test-pre-test model and an experiment team. The Statistical community of the study consisted of all the teenage girls between the ages of 11 and 18 with irresponsible parents or no parents in 24-hour centers covered by the Welfare Organization of Tehran (autumn, 2016). Because the minimum sample size in experiment researchers is 15 for each group [**28**], 15 individuals were selected for each group to calculate the sample size. Afterwards, 20 individuals (n = 20) were selected from each cluster to increase statistical competence and to manage probable drops in participants.

The criteria for entering the present study included the following index:

- Having a conscious tendency to participate.

- Having the ability to take part in sessions and do assignments.

- Being a teenage girl from 24-hour welfare centers in Tehran.

- Being between the ages of 11 and 18.

- Having a minimum education (grade 5 of elementary school).

- Being a psychologically and physically healthy girl with irresponsible parents or no parents. 

The criteria for exiting the study included the following indexes:

- Having no tendency to participate in sessions, more than three sessions of absence during training.

- Not having the ability to take part in sessions and do assignments.

- Having intense mental disorders, having one medical disorder that affected the result of interventions.

- Receiving other psychological or medical treatments that were not part of the study.

- Being younger than 11 or older than 18.

- Not having enough education or being literate. 

The method of performing the research included the visiting of 24-hour Welfare Centers in Tehran that covered girls with irresponsible parents or no parents, and after ensuring the necessary criteria for entering and exiting sessions, 40 girls were randomly selected as a sample, each group having 20 members. Moreover, the girls received explanations about this research, therapy sessions, and research questionnaires; and when they accepted to participate in the sessions, each person was randomly replaced in the control or the experiment group. Before performing the research, the ethical principles of the research were observed as well as the presence of girls who had irresponsible parents or no parents was ensured, the girls receiving explanations about the research and its positive effects. Afterwards, they were asked to announce whether they wanted to participate or not consciously. Next, the experiment group had six sessions (based on six stages of Dezorila and Glad Frid’s pattern) of group training for problem solving; however, the check team did not get any interventions. Finally, both teams were post-tested. The protocol of problem-solving training sessions is presented in **Table 1**.

The tools used in this study included a demography questionnaire, Rozenberg’s self-esteem scale [**3**,**11**], and a behavioral adjustment questionnaire [**1**].

Demography questionnaire: Scholars were provided this questionnaire to receive personal information from respondents. In this questionnaire, there were items such as respondents’ age and education. 

Rozenberg’s Self-esteem order: Rozenberg’s self-esteem order that was made by Rosenberg in 1965 measures the general self-esteem and personal value. This scale included ten common objects that measured life pleasure and having a great feeling of oneself [**12**]. Based on the Bornet & Right (2002), Rozenberg’s self-esteem order (SER) is one of the most popular orders for measuring self-esteem; and it is a well-recognized order, since, for self-esteem, it employs a theory similar to the one presented in mental theories about oneself. SER was formed to give a general image of positive and negative attitudes to oneself (Rosenberg, 1979). Using Cronbach’s alpha, Rosenberg described the safety of the questionnaire to be 0.89. This order has determined relationship coefficients similar to Cooper Smith’s self-esteem survey (SEI); and when estimating the levels of self-esteem, it has excellent efficacy (Grifits et al., 1997). To perform this test, an order has to be allocated to respondents and the respondents are requested to declare their opinion by choosing items such as “I agree” or “I disagree”. Rosenberg stated the reformation of the order to be 0.9 and the scalability of the order to be 0.7 [**22**]. In the first turn, Cronbach’s alpha factor for this order was 0.87 for males and 0.86 for females; and, in the second round, it was 0.88 for males and 0.87 for females. The relationship of retest was in a range of 0.82 and 0.88, and the internal flexibility coefficient or Cronbach’s alpha was between of 0.77 and 0.88 (Newton et al., 1999). This order had acceptable validity (0.77). 

Behavioral adjustment questionnaire: This questionnaire, which has 78 items, was designed and normalized by Sajedi in 1996, including three general dimensions: emotion (questions 1-6, 13, 16, 21-37, 40, 41, 43, 47-52), cognition (questions 8-12, 14, 15, 17-20, 38, 39, 42, 44-46, 53-59), and education (questions 61-78). Each of the questions was on a six-point Likert scale (always to never). The validity and confirmation of this survey was stated to be effective in various studies. Using Cronbach’s alpha, Sajedi (1996) calculated the validity of the rate and stated it to be 0.91, 0.84, and 0.90 for emotion, cognition, and education, respectively, which were favorable. Also, the validity of the questionnaire was measured by Faramarzi, Asgari, and Taghavi (2013) [**14**]. Using Cronbach’s alpha, internal correlation of this scale was calculated to be 0.77 for emotion, 0.79 for cognition, and 0.78 for education. Also, in a study, using Cronbach’s alpha, Khodapanahi and Khaksarboldaji (2009) [**10**] calculated the reliability of the questionnaire to be 0.95 for the sub-scale of emotion dimension, 0.89 for the cognition sub-dimension, and 0.99 for the education sub-dimension. Additionally, using Cronbach’s alpha, its validity is calculated to be 0.91 for variables, which shows high consistency in the scale. This questionnaire was examined by some experts to determine validity; the validity of the questionnaire was calculated to be 0.86 (Sajedi, 2001) [**14**]. 

To analyze data, SPSS-20 software was used. In a statistical method for analyzing the data obtained from the study in a descriptive statistics level, indexes like average, nominal deviation, frequency, and frequency percentage were used; also within an inferential statistics stage, the tests of a single-variable and multivariate covariance analysis were used. 

**Table 1:** Protocol of problem-solving training sessions based on Dezorila and Glad Frid’s pattern

Session	Subject
First	General orientation, ability to recognize problems, accepting problems as natural and changeable phenomena, believing in the effectiveness of problem solving when facing difficulties, great self-efficacy expectations in order to perform stages, being used to cessation, thinking, and then trying to solve problems.
Second	Definition and formation of problems, gathering all the available information, separating facts from assumptions which need research, analyzing problems, setting real goals.
Third	Producing alternative solutions, determining a spectrum of probable solutions, possibility to select the most effective response from responses.
Fourth	Decision, prediction of probable consequences of each measure, attention to the usefulness of these consequences.
Fifth	Performing solutions, performing selected methods.
Sixth	Review, observation of results obtained from performance and evaluation.

**Research Findings**

The demographic characteristics of the present sample are shown in **Table 2**.

**Table 2:** Demographic characteristics of the present sample

group	age	frequency	frequency percentage
Problem-solving	12	1	6.7
	13	4	20
	14	6	40
	15	6	40
	16	3	13.3
	Mean and standard deviation	14/ 40 ± 1/ 14	
control	12	5	25
	13	1	5
	14	4	20
	15	7	35
	16	2	10
	17	1	5
	Mean and standard deviation	14/ 15 ± 1/ 53	
group	education	frequency	frequency percentage
Problem-solving	Elementary	10	50
	Junior high school	10	50
control	Elementary	11	55
	Junior high school	9	45

**Table 3:** Descriptive statistics for the scores of research variables in the two groups based on pre-test and post-test

component	index	experiment		check	
		Pre	Post	Pre	Post
Self-esteem	Average	2.35	7.70	3.15	3.30
	Nominal deviation	1.22	1.21	0.98	1.03
Emotion dimension	Average	82.05	108.55	92.70	93.01
	Nominal deviation	7.93	13.48	10.96	11.23
Cognition dimension	Average	54.50	112.40	10.37	11.26
	Nominal deviation	5.33	16.22	51.95	52.85
Education dimension	Average	45.60	91.80	51.95	52.85
	Nominal deviation	5.03	20.89	6.72	6.26

As shown in **Table 3**, in the experiment group, the average of self-esteem, emotion, cognition, and education scores in the post-test level has increased compared to the control group. 

**Table 4:** Results of Lon’s test in examining the consistency of variances, self-esteem, and its behavioral adjustment dimensions in the post-test level

variable	stage	F	Degree of freedom 1	Degree of freedom 2	Significance level
Self-esteem	Post-test	0.250	1	38	0.620
Emotion dimension	Post-test	0.384	1	38	0.539
Cognition dimension	Post-test	0.569	1	38	0.455
Education dimension	Post-test	0.434	1	38	0.614

As presented in **Table 4**, the assumption of zero about the equality of variances of both groups in self-esteem and dimensions such as emotion, cognition, and emotion were approved. For example for all the variables of self-esteem and emotion, cognition, and emotion, behavioral adjustment of variances in two groups in the society were equal, with no significant differences. Therefore, following Lon’s pre-hypothesis, it was possible to perform a covariance analysis of results to examine the hypothesis of the study. 

**Table 5:** Results of multivariate covariance analysis on post-test scores by controlling pre-test in self-esteem and dimensions such as emotion, cognition, and cognitive-adaptive education

Test title	value	F	Degree of freedom	Significance level	Squared Eta	competence
Pylayy effect	0.744	34.935	3	0.001	0.744	0.95
Wilks Lambda	0.256	34.935	3	0.001	0.744	0.95
Hotelling effect	8.416	34.935	3	0.001	0.744	0.95
Ray’s largest root	8.416	34.935	3	0.001	0.744	0.95

As shown in **Table 5**, the clear stage of all exams (p > 0.001) showed that at least in one of the related parameters (behavioral adjustment dimensions and self-esteem), there was a clear distinction between the 2 teams. In addition, according to the eta square, 0.74 percent of the observed differences between individuals were related to the effect of independent variable, i.e. intervention method (training problem-solving skills). On the other hand, because the statistical competence was 0.95 percent (greater than 0.80 percent), the sample size was acceptable. Results were connected to the significant difference of each dependent variable presented below.

**Table 6:** Results of multivariate covariance analysis for the examination of the effect of training problem-solving skills on self-esteem and behavioral adjustment dimensions in post-test level

index	Sum of squares	Degree of freedom	Mean of squares	F	Significance level	Eta square
Self-esteem	193.601	1	193.601	152.009	0.001	0.801
Emotion dimension	2418.025	1	2418.025	15.699	0.001	0.292
Cognition dimension	21206.025	1	21206.025	108.701	0.001	0.741
Education dimension	15171.025	1	15171.025	63.774	0.001	0.627

Based on the information presented in **Table 6**, since the significance level was p > 0.001, the assumption that there was a difference of self-esteem, and behavioral adjustment dimensions between the two groups were approved. Also, it was stated that 0.80 percent of the changes in the score of self-esteem, 0.29 percent of changes in the score of emotion, 0.74 percent of changes in the score of cognition, and 0.62 percent of changes in the score of education were due to an independent variable (training problem-solving skills). Therefore, the training problem-solving skills helped increase self-esteem and behavioral adjustment dimensions in students. 

## Conclusion

According to the current research which aimed to examine the effect of training issue-solving experiment on the self-esteem and behavioral adjustment in teenage girls with irresponsible parents or no parents, the findings obtained from analyzing the single-variable and multivariate covariance showed that training problem-solving skills have a significant effect on self-esteem and behavioral adjustment in girls with irresponsible parents or no parents. This finding is in congruence with findings obtained from studies conducted by Mahmudi Rad, Arasteh, Afgheh and Barati (2008) [**19**], Ghanbari Hashemabadi and Shahabi (2009) [**16**], Baghayi Moghadam, Malak Pour, Amiri and Molavi (2012) [**4**], and Botvin & Griffin (2004) [**27**].

Ghanbari Hashemabadi and Shahabi (2009) [**16**] stated that training an important skill like problem-solving and receiving a positive feedback helps increase confidence in the society. Also, Botvin & Griffin (2004) [**27**] reported that training life skills in groups helps decrease unpleasant states such as lack of trust. Because by getting together in a group and feeling that others have the same problems as us, we can use each other’s experiences to confront problems and prevent unpleasant emotions or disorders such as anxiety and stress. Baghayi Moghadam, Malak Pour, Amiri and Molavi (2012) [**4**] also stated that training life skills in groups and placing individuals in a group sharing similar problems have significant impacts on interpersonal relationships, leading to a decrease in anxiety, which helps increase self-confidence. 

Baghayi Moghadam, Malak Pour, Amiri and Molavi (2012) [**4**] also stated that training life skills like problem-solving in groups significantly helps improve self-esteem. By gathering in groups and feeling that others have similar problems with ours, we can share our experiences to confront difficulties. On the other hand, training life skills helps individuals learn more about themselves, know their weaknesses and strengths, solve their problems, and strengthen their positive points, which leads to an increase in confidence.

Moreover, the findings of the present study showed that training problem-solving skills affect adjustment components (emotion, cognition, and education) in teenage girls with irresponsible parents or no parents in Karaj. Findings obtained from a multivariate covariance analysis showed that training problem-solving skills significantly affects adjustment dimensions such as emotion, cognition, and education in teenage girls with irresponsible parents or no parents. This finding is in congruence with results obtained from studies conducted by Moradi and Kalantari (2007) [**20**,**21**], Hajamini et al. (2009) [**7**], Khalatbari, Ghorban Shirudi and Mablaghi (2011) [**15**], and Bapiri et al. (2011) [**2**].

Moradi and Kalantari (2007) [**21**] stated that training life skills like problem solving have as final goal the improvement of interpersonal relationships in social life, which enhances social skills. However, some experts believe that social skills and adjustment are equal and have the same meaning. In conclusion, training life skills and developing social skills lead to an increase in individuals’ adjustment. Hatami and Kawsian (2014) [**6**] stated that teaching life skills, such as problem solving and practicing them in everyday life, help people control their behavior and adjust their social interactions in an adaptive way. Additionally, it helps them understand others and predict that all these things significantly increase the individuals’ adjustment. 

**Acknowledgments**

The authors would like to thank the Welfare Organizations for their help. Additionally, they would also like to thank all the participants in the study.

## References

[1] Aghmohammadi M (2007). Examination of behavioral adjustment in junior high school
students in Behbahan. Psychology BA thesis, Psychology University, and
educative sciences. Islamic Azad University, Malayer Branch.

[2] Bapiri O, Bahamin Gh, Feizollahi Ali (2011). Examining the effect of problem-solving group training on some
psychological characteristics in teenagers committing
suicide. Scientific Journal of Ilam Medical Science University.

[3] Bashlideh K, Yusefi N, Haghighi J, Behrouzi N (2013). Examining the factorial structure of Rozenberg’s self-esteem with
three word-processing forms in students of Shahid Chamran University in
Ahwaz: positive, negative, and half the materials: positive, and the other
half: negative. Educative Psychology Studies.

[4] Baghayi Moghadam G, Malak Pour M, Sholeh A, Hossein M (2012). Effect of training life skills on anxiety, happiness, and anger
control in adolescents with the physical disability. Behavioral Sciences Journal.

[5] Behpazhuh A, Soleimani M, Afrouz Gh, Lavasani M (2011). Effect of training social skills on social adjustment and slow
learners' educational performance. Educational innovations.

[6] Hatami M, Kawsian J (2014). Effect of training life skills on the adjustment of dedicated and
martyred parents’ children. Scholastic Psychology.

[7] Hajamini Z, Ajali A, Fathi Ashtiani A (2009). Effect of training life skills on adolescents’ emotional
reactions. Behavioral Sciences Journal.

[8] Hoseinpour Sh, Rasulzadeh T, Khodapanahi M (2009). Examining the relationship between adjustment levels and
adolescents’ personality traits. Behavioral Sciences Journal.

[9] Khodabakhshi A, Rastak  H, Mansour L (2015). The relationship between nutritional habits with a bodily image
and anxiety in adolescents. Psychotherapy.

[10] Khodapanahi M, Khaksarboldaji  MA (2015). The relationship between religious orientation and psychological
adjustment in students. Psychology Quarterly.

[11] Rajabi GhR, Bohlul  N (2008). Measuring reliability and validity of Rozenberg’s self-esteem of
first-year students in Shahid Chamran University. Educative and Psychological Studies.

[12] Safaralizadeh  F, Partoazam H, Habibpour  Z (2011). Correlation of depression and body mass in female adolescents in
Khoy. Correlation of depression and body mass in female adolescents in
Khoy.

[13] Ashouri  M, Jalilabkenar  S (2013 ). Practical hints for training students with learning
disorders. Exceptional Education.

[14] Faramarzi  S, Asgari  K, Taghavi  F (2013 ). Effect of cognitive-behavioral therapy on the adjustment of high
school students with abnormal grieving. Behavioral Sciences Research Journal.

[15] Ghorbanshiroudi  Sh, Khalatbari  J, Toudar  R, Mablaghi  N, Salehi  M (2011). Comparing the effect of training assertiveness skills with the
effect of training problem-solving methods on adjustment and aggression in
female first-year high-school students. New Findings in Psychology.

[16] Ghanbari Hashemabadi  B, Shahabi  M (2009). Examining the effect of training critical thinking on self-esteem
and problem-solving skills in female high school students. Educative Psychology (Psychology and Educative sciences).

[17] Kazemi A, Fahimi S, Mojtabayi M (2014). Expressing psychological clinical symptoms in adolescents based
on Gary’s personality biological pattern. Medical Sciences University Journal in Mazandaran.

[18] Mojarrad Kahani AH, Ghanavi S (2013). Effect of training communication skills on self-esteem in girls
with physical disabilities. Research on Rehabilitation Sciences.

[19] Mahmudi Rad M, Arasteh HR, Afgheh S, Barati SF (2008). Examining the role of training communication skills and social
problem-solving on self-esteem and intelligence in third-grade elementary
students. Rehabilitation.

[20] Moradi A, Dehnavi S (2013). Comparative examination of the effect of group training for
self-esteem, self-efficacy and motivation to progress on self-esteem women
with physical disabilities. Psychology of Exceptional Individuals.

[21] Moradi A, Kalantari M (2007). Effect of training life skills on women’s mental silhouette, who
have physical disabilities. Studying Exceptional Children.

[22] Makund Hosseini Sh (2014). Examining data incorporated in computer systems of 1480 telephone
center, 123 social emergency, and prevalence of social traumas, and their
classification in Semnan. Welfare Organization plan.

[23] (2009). World Health Organization.

[24] Cooper Smith S (1967). The antecedent of self-esteem.

[25] Frieson  TC (2008). Relationship Between hope and self-esteem in Renal Transplant
Recipients. Transplantation Proceeding.

[26] Kaplan VA, Sadock  BH (2007). Kaplan and Sadock’s Synopsis of Psychiatry: Behavioral
Sciences/Clinical.

[27] Botvin  GJ, Griffin  KW (2004). Life skills training: empirical findings and future
directions. The Journal of Primary Prevention.

[28] Delavar A (2007). Research Methods in Psychology and Educational Sciences.

